# Long-Term Follow-up and Risk of Recurrence of Internal Herniation after Roux-en-Y Gastric Bypass

**DOI:** 10.1007/s11695-023-06653-9

**Published:** 2023-06-02

**Authors:** Hassan Zaigham, Mikael Ekelund, Sara Regnér

**Affiliations:** 1 Surgery Research Unit, Department of Clinical Sciences Malmö, Lund University, Malmö, Sweden; 2 Department of Surgery and Gastroenterology, Skåne University Hospital, Malmö, Sweden

**Keywords:** Bariatric surgery, Gastric bypass, Hernia, Abdominal surgery, Internal hernia, Treatment outcome

## Abstract

**Purpose:**

Internal herniation (IH) is the most common complication after Roux-en-Y gastric bypass surgery (RYGB). Although primary closure has reduced the incidence, recurrences are a continued problem. This study aimed to investigate long-term follow-up and recurrence risk of IH surgery.

**Methods:**

A retrospective cohort study of laparoscopic RYGB operated patients operated for a first IH between April 2012 and April 2015 at Skåne University Hospital in Malmö, Sweden. Status of primary closure of mesenteric gaps, time since RYGB, and findings at IH surgery were retrieved from medical records. Follow-up until December 31st, 2019, included recurrences of IH, number of computed tomography (CT) scans, emergency visits, readmissions, and other acute surgeries.

**Results:**

IH (*n* = 44) occurred almost equally in Petersen’s space (*n* = 24) and beneath the jejunojejunostomy (*n* = 20). Long-term follow-up (median 75 months) of 43 patients registered an IH recurrence rate of 14% (*n* = 6). All recurrences occurred in the other mesenteric gap. One patient suffered a third IH, and one patient had four IH events. During follow-up, 56% (*n* = 24) had ER visits for abdominal pain, 47% (*n* = 20) had ≥ 1 abdominal CT scan, and 40% (*n* = 17) were readmitted. A third of readmitted (6/17) patients suffered a recurrence of internal herniation. Two other patients were readmitted ≥ 10 times for chronic abdominal pain.

**Conclusion:**

Surgery for IH had a low risk of recurrence at the treated mesenteric gap, but a 14% recurrence risk at the other mesenteric gap, emphasizing the importance of carefully investigating weaknesses or gaps at the other mesenteric defect during surgery for IH.

**Graphical Abstract:**

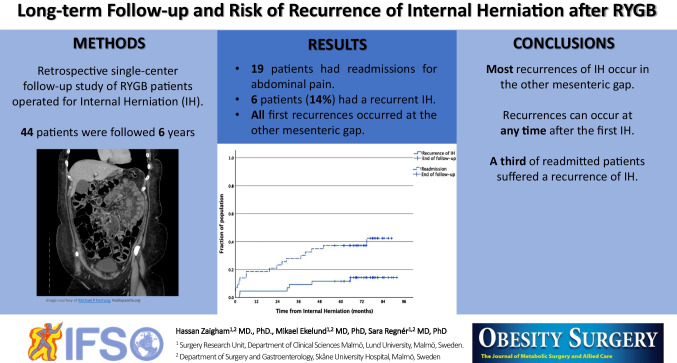

## Background

Internal herniation (IH) is the most common serious surgical complication following laparoscopic Roux-en-Y gastric bypass (RYGB) [1]. Fortunately, the incidence has decreased substantially over time, largely due to primary routine closure of the mesenteric gaps and because examination and closure of open mesenteric gaps are recommended during all laparoscopies [2–4]. However, clinical experience and studies have shown that IH can be a recurring problem with reports of recurrence rates of up to 19% [5]. A recurrence rate of almost one-fifth is unsatisfactory and warrants further investigation of how treatment of internal herniations can improve. Few studies have presented long-term follow-up after surgery for IH, and many studies do not differentiate between surgery for possible intermittent herniations and acute incarcerated herniations.

In Sweden, laparoscopic RYGB constituted over 95% of all bariatric procedures in 2012 but is now on par with sleeve gastrectomies representing nearly half of all bariatric procedures [6, 7]. RYGB is typically performed with an ante-colic, ante-gastric technique. Routine closure of the mesenteric gaps became standard practice across the country in 2010–2011 [8]. The most common closure technique was with an EndoHernia® stapler device (Medtronic, Minneapolis, MA, USA) [9].

During the study period, in the Region of Scania with a population of 1.4 million, bariatric surgery was performed by tax-funded private caregivers on contracts only requiring them to manage complications within the first 30 postoperative days. Annually, 1351–1916 RYGB surgeries were performed in 2012–2015 by private bariatric clinics [10, 11]. While no bariatric surgery was performed at Skåne University Hospital, it had a primary catchment area for acute surgical care of about 500,000, corresponding to about a third of the patients undergoing RYGB surgery in the region. Therefore, late surgical complications to RYGB surgery are mostly handled by general emergency surgeons and only occasionally by surgeons with previous bariatric competency [1].

This single-center retrospective long-term follow-up study aimed to investigate the recurrence rate of patients treated for acute IH and investigate possible surgical factors that may affect the outcome.

## Method

Consecutive patients treated for an acute IH at Skåne University Hospital in Malmö, Sweden, between April 2012 and April 2015 were identified from a previous study cohort of consecutive RYGB patients admitted for acute abdominal pain [1]. IH was defined as surgery for incarcerated bowel in either Petersen’s space or beneath the jejunojejunostomy requiring manual repositioning. Exclusion criteria were previous surgery for internal herniation or a reversal of gastric bypass. Demographic data on sex, age, and total weight loss % since RYGB was noted. Time since RYGB was calculated, and knowledge about the primary closure of the mesenteric gaps was derived from the medical charts. Operation charts for the IH surgery were reviewed to determine whether the surgeon had bariatric competency or not. Bariatric competency was defined as a surgeon with significant experience (> 500 procedures) in bariatric surgery including elective primary and revisional. Albeit vast experience in emergency surgery and laparoscopy, no other surgeon had experience in elective bariatric surgery. It was further noted if the surgery was laparoscopic, converted, or open. The location of the IH in Petersen’s space or beneath the jejunojejunostomy was recorded. Diagnostic laparoscopies with simple closure of mesenteric gaps without evidence of incarcerated bowel were not included. Follow-up information regarding any repeat surgery for IH, surgical readmissions, surgical emergency visits, and acute abdominal computed tomography (CT) scans from any emergency department or surgical ward in our region was accessible and extracted from digital medical records until December 31st, 2019.

### Statistical Analysis

All data were stored and analyzed using SPSS version 28 (IBM Corporation, Armonk, NY, USA). Results are expressed as median with minimum and maximum values. Mann–Whitney *U* non-parametric test was used for group comparisons of continuous values, while Fisher’s exact test was used for the comparison of categorical variables. All group comparisons were unpaired. A two-sided *p*-value of ≤ 0.05 was considered statistically significant.

## Results

During the 3-year inclusion period, 444 acute surgical admissions of RYGB patients were registered. Surgery for incarcerated internal herniation was performed on 44 patients. Five patients were operated on based on their clinical presentation without preoperative imaging. The study population had typical sex distribution for the RYGB population with a female-to-male ratio of 4:1, and the patients suffered an IH at a wide time-interval from their RYGB surgery, including one postoperative event that was readmitted on postoperative day 1 (Table 1). About half of the patients had IH despite closed mesenteric gaps at RYGB surgery, and the most common primary closure method was using clips (18/21). Surgeries for IH were mostly attempted laparoscopically (42/44) but with a high conversion rate of 38% (Table 2). Patients having laparotomies had significantly longer hospital stays (median 4.5 days) compared to those managed laparoscopically (median 2 days, *p* < 0.001). Two surgeons with bariatric competency were available for five surgeries (11%). They had an equally high conversion rate of 40% (2/5). The IHs were evenly distributed between Petersen’s space (*n* = 24) and beneath the jejunojejunostomy (*n* = 20). Operation charts were available for 43/44 patients. Petersen’s space was closed and ensured in all patients, but the mesenteric gap beneath the jejunojejunostomy was not reported to have been inspected in three patients (7%). One patient required a bowel resection and a reconstructed anastomosis. All open mesenteric gaps were closed using running sutures. Non-absorbable sutures were used for 38 patients and absorbable sutures for two patients. Information about suture material was missing for four patients. The reported non-absorbable sutures were Ethibond® (*n* = 31) and Prolene® (*n* = 5) (Johnson & Johnson, New Brunswick, NJ, USA) with brand data missing for two patients. The absorbable sutures Vicryl® and PDS® (Johnson & Johnson, New Brunswick, NJ, USA) were used for one patient each.

**Table 1:** Demographics and data from RYGB for the study cohort

	Median (min–max)	*n*	(%)
Sex			
- Female		35	(80)
- Male		9	(20)
Age (years)	40.2 (20–58)		
BMI (kg/m^2^)^1^	27.5 (19–39)		
% Total Weight Loss^2^	33.9 (8–55)		
Time since RYGB (months)	17.9 (0–63)^3^		
Primary closure of mesenteric gaps			
- Closed		21	(48)
- Not closed		12	(27)
- Unknown		11	(25)

*BMI* body mass index, *RYGB* Roux-en-Y gastric bypass

^1^Missing data for 5 patients (11%)

^2^Missing data for 11 patients (25%)

^3^One patient was readmitted on the 1st postoperative day following RYGB

**Table 2:** Results of internal herniation surgery

	*n*	(%)
Surgical access		
- Laparoscopic	26	(59)
- Converted	16	(36)
- Laparotomy	2	(5)
On call surgery	25	(57)
Bariatric competency	5	(12)
Internal herniation in		
- Petersen’s space	24	(55)
- Beneath jejunojejunostomy	20	(45)
Closure method		
- Non-absorbable suture	38	(86)
- Absorbable suture	2	(5)
- Undocumented suture	4	(9)

### Long-Term Follow-up

**Fig. 1:**
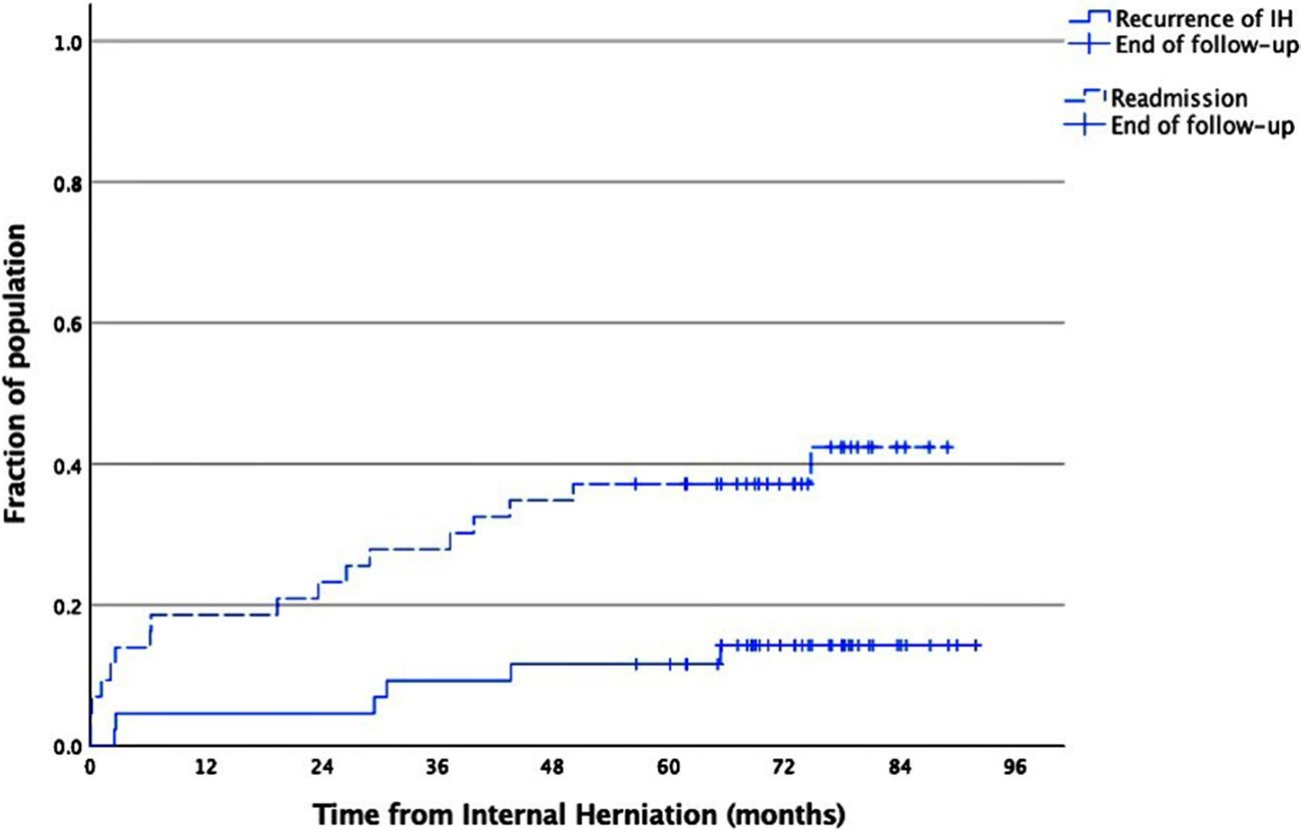
The Kaplan–Meier cumulative incidence plot of readmissions and recurrences of internal herniation after treatment for internal herniation

Long-term follow-up of in median of 74.8 months was available for 43/44 (98%) patients treated for acute internal herniation (Table 3). One patient had moved abroad and was thus lost to follow-up. Six patients (14%) required surgery for recurrent IHs. All first recurrences occurred in the other mesenteric gap compared to the first internal hernia, i.e., in Petersen’s space if the first herniation was beneath the jejunojejunostomy and vice versa. One patient had a third IH while another patient suffered four IHs, all occurring in Petersen’s space. None of the five patients operated on by surgeons with bariatric competency had a recurrence of IH. Recurrences occurred irrespective of previous open (2/17) or laparoscopic surgery (4/26) for IH. Recurrent IHs occurred between 2.5 and 65 months from the first operation for IH with a median time of 30 months (Fig. 1). The three patients that did not have a reported inspection of the mesenteric gap beneath the jejunojejunostomy did not suffer any recurrences during the follow-up. All recurrences occurred at a mesenteric defect that was inspected during the first IH surgery. Four patients had undergone secondary closures of the defects where they subsequently suffered a recurrence, while two patients suffered recurrences in Petersen’s space that had been assessed as closed and not needing reinforcement.

**Table 3:** Follow-up data for patients operated for internal herniation

	Median (min–max)	*n*	(%)
Patients observed		44	(100)
Lost to follow-up		1	(2)
Follow-up time (months)	74.8 (57–92)		
Computed tomography scans			
- 1		5	(12)
- 2		6	(14)
- ≥ 3		9	(21)
Emergency department visit(s)		24	(56)
Readmissions		17	(40)
Time to readmission (months)	6.8 (0.1–50)		
Internal herniation recurrences		6	(14)
- in Petersen’s space		4	(9)
- beneath jejunojejunostomy		2	(5)
Time to internal herniation recurrence (months)	30 (2.5–65)		
Multiple internal herniation recurrences		2	(5)
Surgery for			
- other RYGB complication		3	(7)
- other acute abdominal condition		4	(9)

RYGB = Roux-en-Y Gastric Bypass

More than half of the patients (24/43) had recurrent emergency department (ED) visits for abdominal pain. Nine (21%) patients had three or more visits for acute abdominal pain. Seventeen (40%) patients had at least one readmission for abdominal pain. In total, 62 readmissions were recorded. The Kaplan–Meier survival plot (Fig. 1) illustrates that about a third of the readmitted patients (6/17) suffered a recurrence of internal herniation. Twenty patients had a total of 85 CT scans during the follow-up period, with three patients having 30 of these scans. Two (4%) patients had ≥ 20 ED visits and 10 and 17 readmissions respectively, neither of whom had a recurrence of an internal herniation. One of them instead first underwent two surgeries for small bowel obstruction caused by an intussusception and then another exploratory laparotomy for the suspicion of another intussusception but without conclusive findings. The other patient had repeated inconclusive diagnostic laparoscopies and then underwent a revision of the jejunojejunostomy. Both of these patients were diagnosed with chronic abdominal pain.

Three patients with recurrences of internal herniation also had separate readmissions and surgeries for appendicitis, cholecystitis, and a sigmoid volvulus respectively. One patient was readmitted and operated on for adhesive small bowel obstruction as a complication to the RYGB. Another patient underwent a subacute cholecystectomy during readmission.

## Discussion

The current study showed an internal herniation recurrence risk of 14% over a 6-year follow-up which is lower than previously published data, but still higher than the reported risk of primary IH [3, 5, 12]. About a third of all readmitted patients (6/17) suffered a recurrence of internal herniation, proving the importance of maintaining a high degree of suspicion in readmitted patients. However, as patients with chronic abdominal pain had multiple readmissions, the fraction of internal herniation out of all readmissions was only 6/62 (10%). This figure corresponded well with the proportion in the observed population, as 44 patients suffered an internal herniation among 444 admissions for acute abdominal pain during the 3-year inclusion period.

Interestingly, all recurrences occurred in the other mesenteric gap compared to the first IH indicating the importance of carefully investigating the other site for weaknesses or gaps that need closing or tightening. Furthermore, bariatric competency ensured that no recurrences occurred during the observed follow-up period, which underlines the importance of bariatric competency being available for reoperations in this cohort. A high conversion rate of 41% demonstrated the challenge of treating incarcerated bowel in internal hernias with minimally invasive surgery. A couple of previous studies have reported similarly high conversion rates of 33–40%, while most other studies report lower rates [13, 14]. The high conversation rate in our study may be explained by the strict inclusion of patients with truly incarcerated bowel in contrast to studies reporting symptom relief following the closure of mesenteric gaps in patients with intermittent herniations.

The fact that internal herniations and recurrent IHs can occur at any time following RYGB is well known [5]. The challenge is to reduce the incidence further. The primary incidence of internal herniation has fallen after routine closure of mesenteric gaps at RYGB surgery [2, 3]. Secondary closure also needs to be performed securely to ensure a low risk of recurrence.

The frequency of CT scans was higher than previously reported as 47% of patients had at least one CT scan during our 75-month follow-up compared to 40% reported by Sandvik et al. in a similar setting but for a 100-month follow-up [15]. This may be explained by the general trend toward more frequent scans due to greater access and a lower referral threshold. The high number of ER visits and readmissions for abdominal pain are well-known complications of the Roux-en-Y gastric bypass [1, 16]. Two patients suffered from chronic abdominal pain requiring multiple CT scans, ER visits, and admissions. Chronic pain after RYGB is an unfortunate and difficult condition to treat that has been reported at higher rates in several studies [17–19].

As a single-center study, a drawback of this study is the relatively few patients included. For the inclusion period of 3 years, however, the number of patients treated for internal herniation is high and explained by a high frequency of RYGB-operated patients in the population served by the hospital. Also, the follow-up percentage of 98% is excellent owing to accessible electronic medical charts for the whole Scania region covering all emergency departments and surgical wards for our region’s population. A multi-center study would benefit from being able to include more patients but could result in a lower follow-up percentage depending on the availability of follow-up data. A strength of the current study was the strict inclusion of only incarcerated herniations which ensured that there was no case mix with patients with asymptomatic open mesenteric gaps. The current acute abdominal pain may not always respond to the routine closure of open mesenteric gaps at an otherwise unremarkable exploratory laparoscopy. The problem of symptom relief after the closure of mesenteric defects was addressed by Wijngaarden et al. [20]. However, defining the population for comparison is difficult, making it challenging to draw accurate comparisons between studies.

## Conclusion

Surgery for IH showed a low risk of recurrence in the treated mesenteric gap but a high risk of recurrence in the other mesenteric gap. The results emphasize the importance of carefully investigating weaknesses or gaps in the other mesenteric gap at the surgery for IH to reduce the risk of recurrence.
